# Reclaiming to Brackish Wetlands in the Alberta Oil Sands: Comparison of Responses to Sodium Concentrations by *Carex atherodes* and *Carex aquatilis*

**DOI:** 10.3390/plants10081511

**Published:** 2021-07-23

**Authors:** Lilyan C. Glaeser, Melissa House, Dale H. Vitt

**Affiliations:** School of Biological Sciences, Plant Biology, Southern Illinois University, Carbondale, IL 62901, USA; mhouse@siu.edu (M.H.); dvitt@siu.edu (D.H.V.)

**Keywords:** Alberta, boreal, *Carex aquatilis*, *Carex atherodes*, oil sands reclamation, Sandhill Wetland, sodium tolerance, wetland

## Abstract

The variation in sodium concentrations in waters of natural fens and marshes on the western Canadian landscape provides a background for choosing the appropriate plants for wetland reclamation. Broad tolerances to salinity are especially important for reclamation trials on saline-rich ‘in-pits’ that were left from open-pit oil sands mining. One such species, *Carex aquatilis*, has been identified as a key species in early reclamation attempts; however, at the Sandhill Wetland on the Syncrude Canada oil sands lease, this species has aggressively colonized, dominating parts of the wetland and limiting species diversity. A second species, also widespread on natural lake shores and marshes, is *Carex atherodes*, with field observations suggesting a broad tolerance to salinity. Here, we examine the responses of this species to a series of sodium concentrations and compare these to those of *C. aquatilis.* In particular, we addressed three questions: (1) How do structural attributes of *C. atherodes* respond to a series of Na^+^ concentration treatments? (2) Are different structural responses related to the functional attributes of photosynthesis, stomatal conductance, and/or transpiration rate? (3) How do these responses compare to those of *C. aquatilis?* We implemented a phytotron experiment to test the responses of these two species to either five or six concentrations of sodium, ranging from 20 to 3000 mg Na^+^ L^−1^. In general, structural responses of *C. atherodes* did not differ between 50 and 789 mg Na^+^ L^−1^, while performances of all attributes were reduced at 1407 mg L^−1^. Physiological attributes had high variation, but also had reduced performances at similar treatment levels. In comparison, a clear threshold was present for structural attributes in *Carex aquatilis* between 1650 and 2148 mg Na^+^ L^−1^, while physiological attributes were reduced between 1035 to 1650 mg Na^+^ L^−1^. These responses from *C. aquatilis* were similar to those previously reported. Na^+^ concentrations in porewater at the Sandhill Wetland in 2019 reached as high as 1200 mg Na^+^ L^−1^, with natural subsaline and sodic sites ranging much higher. Although all of the plants in the treatments remained viable at the end of the experiment, these results indicate that Na^+^ concentrations above 1500–2000 mg Na^+^ L^−1^ may inhibit the growth of these two species and decrease their competitive abilities.

## 1. Introduction

Oil sand deposits lie under 141,000 km^2^ of the landscape of Alberta, Canada and in 1967, commercial oil sands mining began in northeastern Alberta [1]. Over the past 50 years or so, mining has continued to increase, reaching an oil sands production of 171,084,241 m^3^ (1.1 billion bbl) in 2019 [2]. One method of oil sands extraction is open-pit mining, which accounts for about 20% of mining operations and involves the removal of vegetation and surficial deposits in order to access the oil sands deposits containing bitumen [3]. After mining operations are concluded, these large-scale depressions, or in-pits, are refilled with a variety of tailings and process waters that have relatively high concentrations of cations and anions [4,5,6]. Reclamation of in-pit deposits is legislatively mandated to a return to equivalent land capability [7], and these sites include areas of upland and wetland vegetation that provide a new physical landscape [8].

Peatlands (bogs and fens) cover about 27% of the Oil Sands Administrative Area [9], with nonpeat-forming wetlands (marshes and swamps) being only a minor component [10]. Most abundant on the landscape are rich fens that have pore water chemistries dominated by divalent cations, with Na^+^ concentrations less than 10 mg L^−1^ and Ca^2+^ concentrations less than 100 mg L^−1^ [11]. Brackish and saline marshes are less frequent on the landscape, with these sites having quite different water chemistries (Na^+^ ranging from 654 to 2831 mg L^−1^ and Ca^2+^ from 35 to 224 mg L^−1^) [12,13,14,15,16]. Currently, there are wetland reclamation projects in the Athabasca Oil Sands Region, but the difficulties and novelty of creating new complex systems that emulate natural systems have hindered concrete protocols and methods [3,17]. In early plant establishment, site wetness and chemistry were recognized as the most important limiting factors, and early experimental wetland sites such as Sandhill Wetland were engineered to mitigate these variables [18]. However, the salinity (especially Na^+^) of oil sands landscapes is considerably higher than what is typical of both bogs and fens [12,16], and depending on its severity, high Na^+^ concentrations can provide a harsh limiting environment for many plants [19,20]. Understanding how desired plants will respond to increased Na^+^ concentrations is crucial to successful reclamation.

Purdy et al. [13] described the plant communities of Alberta’s boreal landscape along salinity gradients as potential models for oil sands reclamation. Plant species that had an affinity for flooded and wet meadow communities as well as for strongly to slightly saline soils included *Carex atherodes*, *Scolochloa festucacea*, *Scirpus paludosus*, and *Triglochin maritima* [13]. Furthermore, *Carex atherodes* was not impacted by the process water of oil sands activity in northwestern Canada after two growing seasons of irrigation in a greenhouse, with up to approximately 569 mg Na^+^ L^−1^ [21].

*Carex atherodes* is circumpolar in distribution, occurring from the arctic and ranging as far south as Arizona and New Mexico in the west, and Missouri and Virginia in the east [22]. It frequently occurs in wet meadows, lake shores, fens, ponds, and marshes [23,24]. Trites and Bayley [16] reported this species from a number of slightly brackish (sites with electrical conductance (EC) between 0.5 and 2.0 mS) to moderately brackish (sites with EC from 2.0 to 5.0 mS) marshes in Alberta, including sites with EC from 0.5 to 5.7 mS cm^−1^. The species did not occur in brackish (sites with EC 5.0–15.0 mS) or subsaline sites (those with EC 15–45.0 mS) [25]. The natural occurrences of *C. atherodes* in marshes and lake shores [26,27], many with brackish water chemistries [28,29], suggest that this species may provide a key component to the vegetation of sites with moderate to high concentrations of Na^+^, and for sites where Na^+^ levels exceed those tolerated by other sedges (e.g., *C. aquatilis*). Just as widespread but occurring commonly in fens is *C. aquatilis*, a species previously examined for habitat limitations [30] and tolerances to Na^+^ [31]. Compared to *C. aquatilis*, *C. atherodes* is taller (up to 1.2 m tall vs. 1.0 m for *C. aquatilis*, with more numerous, broader leaves (3–12 mm wide vs. 2.5 mm for *C. aquatilis*) [24].

Three years after wet-up, Vitt et al. [32] described three plant assemblages as dominant on Sandhill Wetland. The plant assemblage that occurs in areas with both intermediate water levels and salinity continues to be dominated by *C. aquatilis*, to the extent of excluding many sub-dominant plant species, including ground layer bryophytes. After seven years of plant development, it is apparent that future reclamations should include additional species that are of similar size and aggressiveness to *C. aquatilis*. Based on field observations, *Carex atherodes* might serve this purpose if it has similar responses to salinity as *C. aquatilis*. The objectives of this study was to further understand the responses to increasing Na^+^ concentrations of *C. atherodes*. In particular, we addressed these three questions:

1. How do the structural attributes of *C. atherodes* respond to a series of Na^+^ concentration treatments that are present or expected at future in-pit reclamation sites in the Alberta oil sands region?
2. Are different structural responses related to functional attributes of chlorophyll, photosynthesis, stomatal conductance, and/or transpiration rate?
3. How do these responses compare to those of *C. aquatilis*?

## 2. Results

### 2.1. Structural Attributes

#### 2.1.1. Biomass

**Figure 1:**
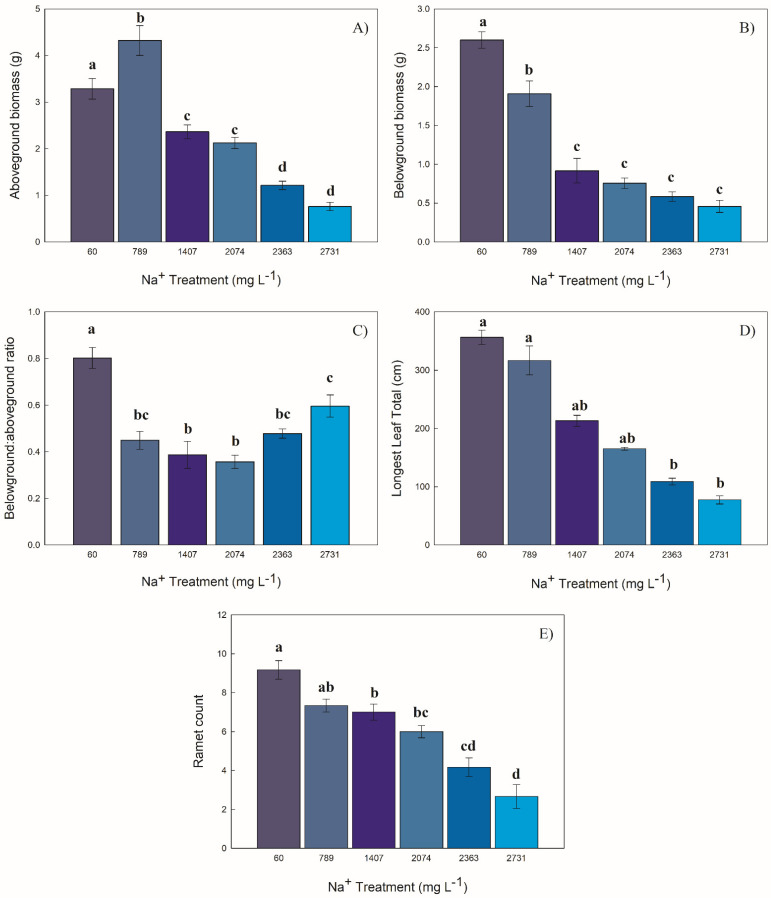
Responses (as mean ± S.E.) of *Carex atherodes* to concentrations of sodium. (**A**) aboveground biomass (F = 67.54, *p* < 0.001), (**B**) belowground biomass (F = 63.46, *p* < 0.001), (**C**) ratio of belowground biomass to aboveground biomass (F = 16.81, *p* < 0.001), (**D**) sum of longest leaves (H = 29.81, *p* < 0.001), (**E**) number of ramets (F = 27.00, *p* < 0.001). Different letters indicate significantly different values between treatments (F = Tukey’s pairwise post hoc test at *p* ≤ 0.05 or H = Dunn’s pairwise post hoc comparison at *p* ≤ 0.05). All data from end of experiment.

Aboveground biomass was different among treatments. The highest biomass was produced by *C. atherodes* exposed to 789 mg Na^+^ L^−1^ (averaging 4.32 g), which decreased dramatically with increasing sodium exposure (Figure 1A). Plants exposed to 789 mg Na^+^ L^−1^ produced 1.8 times more aboveground biomass than plants exposed to 1407 mg Na^+^ L^−1^ and 5.6 times more aboveground biomass than plants exposed to 2731 mg Na^+^ L^−1^.

Belowground biomass production was also different among treatments. The highest biomass was produced by *C. atherodes* exposed to 60 mg Na^+^ L^−1^ (averaging 2.59 g), then decreased, most dramatically after 789 mg Na^+^ L^−1^, with increasing sodium exposure (Figure 1B). Plants exposed to 60 mg Na^+^ L^−1^ produced 1.36 times more belowground biomass than plants exposed to 789 mg Na^+^ L^−1^; however, the latter produced 2.08 times more below ground biomass than plants exposed to 1407 mg Na^+^ L^−1^.

The belowground:aboveground biomass ratio was different among treatments. The lowest treatment, 60 mg Na^+^ L^−1^, had the greatest belowground:aboveground ratio, with the 1407 and 2074 mg Na^+^ L^−1^ treatments having the lowest ratio. Although all treatments produced a greater amount of aboveground biomass than belowground biomass (Figure 1C).

#### 2.1.2. Longest Leaf Length

Among the six treatments, the summed lengths of the longest leaves from the original plant and its ramets varied from 61.1 cm to 401 cm. Sodium treatments had an effect on the longest leaf length, with a steady decrease in length in the 60 to 2731 mg Na^+^ L^−1^ treatments (Figure 1D). *Carex atherodes* leaves were 2.1 to 6.6 times greater in the treatment with 60 and 789 mg Na^+^ L^−1^ than in the 2731 mg Na^+^ L^−1^ treatment.

#### 2.1.3. Ramet Count

*Carex atherodes* produced between one and eleven ramets per individual plant. Sodium treatments had an effect on the number of ramets produced, steadily decreasing with increased sodium (Figure 1E). On average, the plants produced about one less ramet for each treatment increase in sodium (60 mg Na^+^ L^−1^ = 9 ramets; 789 mg Na^+^ L^−1^ = 7.3; 1407 mg Na^+^ L^−1^ = 7; 2074 mg Na^+^ L^−1^ = 6; 2363 mg Na^+^ L^−1^ = 4.2; 2731 mg Na^+^ L^−1^ = 2.6).

### 2.2. Chlorophyll Content

Chlorophyll content ranged from 17 to 38 and was different among sodium treatments (df = 5, H = 35.482, *p* < 0.001). Plants of 60, 789, 1407, 2074, and 2363 mg Na^+^ L^−1^ treatments were similar; plants of 2731 mg Na^+^ L^−1^ were different from those of 60, 789, and 2074 mg Na^+^ L^−1^ treatments.

### 2.3. Functional Attributes

#### 2.3.1. Photosynthetic Rate

**Figure 2:**
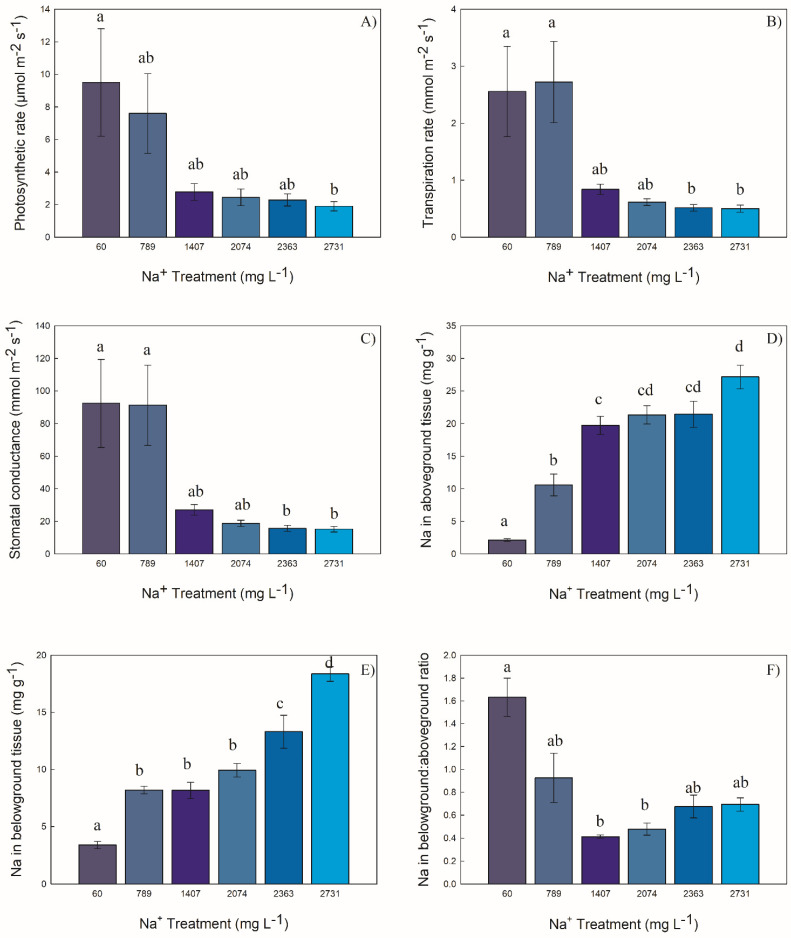
Responses (as mean ± S.E.) of *Carex atherodes* to concentrations of sodium. (**A**) photosynthetic rate (H = 18.80, *p* = 0.002), (**B**) transpiration rate (H = 30.18, *p* < 0.001), (**C**) stomatal conductance (H = 31.31, *p* < 0.001, (**D**) sodium in aboveground tissue (F = 35.91, *p* < 0.001), (**E**) sodium in belowground tissue (F = 54.02, *p* < 0.001); (**F**) ratio of belowground to aboveground tissue concentration of sodium (H = 21.06, *p* < 0.001). Different letters indicate significantly different values between treatments (F = Tukey’s pairwise post hoc test at *p* ≤ 0.05 or H = Dunn’s pairwise post hoc comparison at *p* ≤ 0.05). All data from end of experiment.

Photosynthetic rate was different among the sodium treatments, decreasing with increased sodium exposure. *Carex atherodes* exposed to 60 mg Na^+^ L^−1^ had photosynthetic rates five times greater than those exposed to 2731 mg Na^+^ L^−1^ (Figure 2A).

#### 2.3.2. Transpiration Rate

Transpiration rate was different among the sodium treatments. The two lowest treatments (60 and 789 mg Na^+^ L^−1^) had plants with rates 4.9 times greater than the two highest treatments (2363 and 2731 mg Na^+^ L^−1^) (Figure 2B). Stomatal conductance was correlated with the transpiration rate (*p* < 0.001, R^2^ = 0.99) and the photosynthesis rate (*p* < 0.001, R^2^ = 0.79).

#### 2.3.3. Stomatal Conductance

Stomatal conductance was different among the sodium treatments. The two lowest treatments (60 and 789 mg Na^+^ L^−1^) had plants with rates 5.8 times greater than the two highest treatments (2363 and 2731 mg Na^+^ L^−1^) (Figure 2C).

#### 2.3.4. Concentration of Na in Aboveground and Belowground Biomass

The concentration of Na in aboveground biomass was different among treatments. Plants exposed to the 789 mg Na^+^ L^−1^ sodium treatment had five times the concentration of Na than the lowest concentration (60 mg Na^+^ L^−1^), 10.6 mg Na g^−1^, and 2.1 mg Na g^−1^. The greatest concentration was in *C. atherodes* exposed to 2731 mg Na^+^ L^−1^ (averaging 27.16 mg g^−1^) (Figure 2D).

The concentration of Na in belowground biomass was different among treatments (Figure 2E). The Na concentration increased two-fold from the lowest treatment (60 mg Na^+^ L^−1^) to the next three treatments (789 mg Na^+^ L^−1^, 1407 mg Na^+^ L^−1^, and 2074 mg Na^+^ L^−1^), three-fold in the 2363 mg Na^+^ L^−1^ treatment, and six-fold for the highest treatment (2731 mg Na^+^ L^−1^).

The concentration of Na in the belowground:aboveground biomass ratio was different among treatments. Only the lowest treatment, 60 mg Na^+^ L^−1^, had a higher concentration of Na in the roots than the shoots. The 1407 and 2074 mg Na^+^ L^−1^ treatments were about one-fourth the ratio of the lowest treatment (Figure 2F).

#### 2.3.5. Comparison to *Carex aquatilis*

Under control conditions (40 mg Na^+^ L^−1^), the aboveground biomass of *Carex aquatilis* averaged 2.1 g, belowground biomass was at 2.3 g, about 36% and 12%, respectively, lower than those of *C. atherodes* (Table 1). In the 1035 mg Na^+^ L^−1^ treatment, *C. aquatilis*’ aboveground biomass decreased by 28% and its belowground biomass decreased by 43%. Comparatively, in the 789 mg Na^+^ L^−1^ treatment, the aboveground biomass of *C. atherodes* increased by 24% with a decrease of 27% for its belowground biomass. Photosynthesis of *C. aquatilis* under control conditions averaged 7.2 µmol m^−2^ s^−1^, compared to 9.7 µmol m^−2^ s^−1^ for *C. atherodes*; stomatal conductance was 68 mmol m^−2^ s^−1^ for *C. aquatilis* and 96 mmol m^−2^ s^−1^ for C. *atherodes*. Photosynthesis and stomatal conductance both decreased dramatically at 1035 mg Na^+^ L^−1^ (37% and 59%, respectively, with significant differences at 1650 mg Na^+^ L^−1^). Comparatively, photosynthesis of *C. atherodes* decreased only by 20% (with no change for stomatal conductance) at 789 mg Na^+^ L^−1^. At 1407 mg Na^+^ L^−1^, photosynthesis had decreased by 71% and stomatal conductance by 71%.

## 3. Discussion

### 3.1. Variation in Sodium at Natural Sites and at Sandhill Wetland

**Figure 3:**
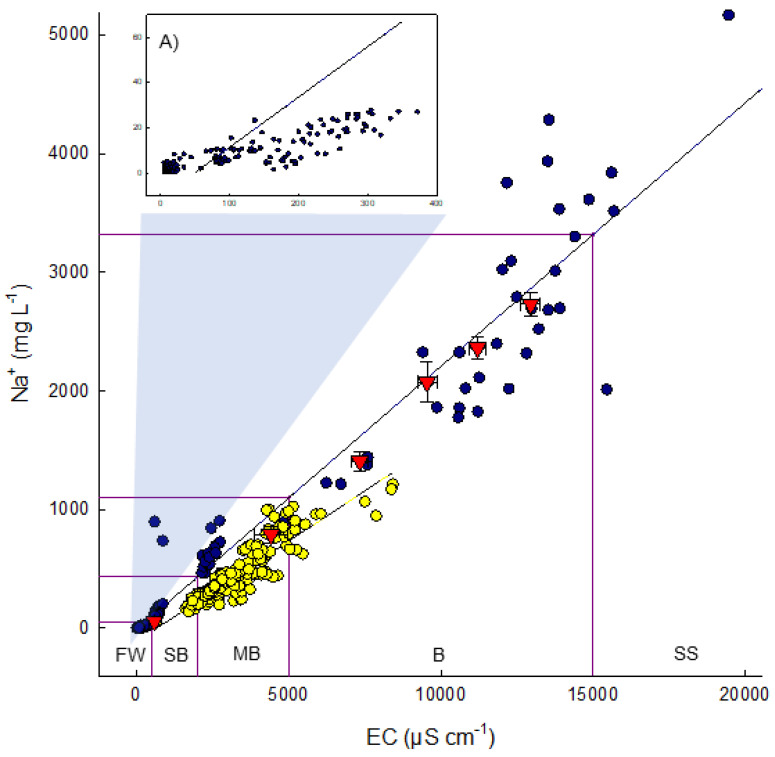
Relationship of Na^+^ to electrical conductance for surface water samples taken from natural subsaline and saline wetlands (blue), samples from Sandhill Wetland (yellow), mean ± S.E. values of concentrations from the six Na^+^ treatments in this study (red triangles). Boxes are wetland classifications: FW—Freshwater (EC < 500), SB—Slightly brackish (EC = 500–2000), MB—Moderately brackish (EC = 2000–5000), B—Brackish (EC = 5000–15,000), SS—Subsaline (EC = 15,000–45,000) [25]. Regression: Na^+^ = 0.22*EC − 10.73. (**A**) Inset shows surface water samples from natural fens expanded. Data from natural sites taken from 2017 to 2018 from [12], and from Sandhill Wetland from 2019 [36].

The natural rich fens of boreal Alberta are dominated by a small suite of *Carex* species, including *C. aquatilis*, *C. chordorrhiza*, *C. diandra*, *C. lasiocarpa*, and/or *C. limosa* [12,33,34,35]. Associated with these species is porewater chemistry that is relatively high in divalent cations (Ca^2+^, Mg^2+^) and low in Na^+^. Considerable variation in electrical conductance (EC) results from variation in divalent cations, with Na^+^ providing only a small fraction of charge influencing EC (Figure 3A). In comparison, shallow marshes (both fresh and brackish), lake shores, open riparian zones, and meadows have a flora composed of *Carex aquatilis*, *C atherodes*, *C. rostrata (s.l.)*, the grass *Calamagrostis canadensis*, and/or *Typha latifolia* [36]. The porewater chemistry of these site types is comparatively higher in Na^+^, leading to brackish and eventually to saline and sodic wetlands. Sodium concentrations along the brackish-saline-sodic gradient are strongly associated with EC and can attain high values (Figure 3). Porewater chemistry at Sandhill Wetland has sodium concentrations that have increased steadily over the first seven years since wet-up, with the highest concentrations of Na^+^ recorded in 2019 at 1646 mg L^−1^ and an overall site mean of 496 mg L^−1^ [37]. These concentrations of sodium far exceed those present in natural fens of the region (Figure 3). The selection of foundation species that respond to brackish/saline water chemistries is a key component in the reclamation of in-pit deposits.

### 3.2. Carex atherodes—Responses of Structural and Functional Attributes

*Carex atherodes* responded with decreased performance in most structural attributes above a treatment of 789 mg Na^+^ L^−1^, with significant differences manifested at a treatment of 1407 mg Na^+^ L^−1^. Structural attributes deceased between 35 and 75% at 1407 mg Na^+^ L^−1^. In comparison, functional attributes were different only at higher treatments, almost certainly due to high variation in the 60 and 789 mg Na^+^ L^−1^ treatments and the lack of variation in the higher treatments. Functional attributes above 1407 mg Na^+^ L^−1^ were remarkably stable with consistently low responses. Photosynthetic rates were strongly associated with the transpiration rate and stomatal conductance, and all of these attributes decreased over four times at a treatment of 1407 mg Na^+^ L^−1^; however, significant decreases were not present until a treatment of 2383 or 2731 mg Na^+^ L^−1^. Sodium in belowground tissues were similar until a treatment level of 2383 mg Na^+^ L^−1^, but above ground tissue concentrations increased steadily over the six treatments. Sodium concentrations in both belowground and aboveground tissues steadily increased as treatment levels rose, with a strong decrease in below-to-aboveground ratios at a treatment of 1407 mg Na^+^ L^−1^, suggesting the saturation of the belowground biomass that resulted in higher aboveground Na tissue concentrations.

### 3.3. Comparison to Carex Aquatilis

*Carex atherodes* produced about 34% more overall biomass than *C. aquatilis* under control conditions; likewise, the photosynthesis rate was 37% higher for *C. atherodes*. Under field conditions, *C. atherodes* is a much more robust plant, both in terms of height and leaf width. At the lowest treatment level (789 mg Na^+^ L^−1^), *C. atherodes* increased aboveground biomass and decreased belowground biomass, suggesting some tolerance of aboveground tissue to increased Na^+^ concentrations. Comparatively, at a slightly higher treatment (1035 mg Na^+^ L^−1^), the biomass of *C. aquatilis* decreased by 30%, with significant differences in responses only present above the 2148 mg Na^+^ L^−1^ treatment. Chlorophyll contents (measured using a SPAD meter) of the two species were similar (in the range of 17–38 for *C. atherodes* and 26–34 for *C. aquatilis*), both exhibiting reduced numbers above ca. 2000 mg Na^+^ L^−1^. All three functional responses produced a reduced performance above the 1035 mg Na^+^ L^−1^ treatment. Although our objectives did not include an examination of the mechanisms of salt tolerance, the concentrations of Na in both the aboveground and belowground tissues of *C. atherodes* suggest a different mechanism from that of *C. aquatilis.* In this mechanism, there is some evidence of salt being concentrated in the root tissue until very high concentrations, where root biomass is much reduced. Previous experiments concluded that *C. aquatilis* had reduced performance above a treatment of 1079 mg Na^+^ L^−1^, which manifested in a treatment of 2354 mg Na^+^ L^−1^, with significant differences in the same structural and functional characteristics [38]. Similar to *C. aquatilis* [38], the transpiration rates of *C. atherodes* are highly correlated with stomatal conductance, indicating a similar relationship to reduced photosynthesis in the two species. The current trials suggest that we can refine the tolerance of *C. aquatilis* to Na^+^, with reduced performance above a 1650 mg Na^+^ L^−1^ treatment and evident at 2148 mg Na^+^ L^−1^. Comparatively, *C. atherodes* showed reduced performance in most attributes above 789 mg Na^+^ L^−1^, with significant differences manifested at a treatment of 1407 mg Na^+^ L^−1^. In similar trials, Koropchak and Vitt [39] found decreased survivorship and biomass of *T. latifolia* at 600 mg Na^+^ L^−1^, while Glaeser et al. [40] found decreased performances and biomass of *Beckmannia syzigachne* after 850 mg Na^+^ L^−1^. The higher tolerances of both species of *Carex* indicate that these two species may provide the key ingredients to the vegetative recovery of oil sands sites with brackish waters.

## 4. Materials and Methods

We used water chemistry collected at Sandhill Wetland in 2019 to explore in-pit reclamation conditions seven years post-wet-up. The 58 ha Sandhill Watershed is an experimental site on a formerly mined-out in-pit located on Syncrude Canada Ltd. oil sands lease at 57.040° N, 111.596° W at 310 m elevation [18]. Its construction consisted of backfilling the 60–100 m deep in-pit with composite and pure sand tailings between 1999 and 2008. Ten meters of sand were mechanically placed, which shaped the present-day watershed. The 17 ha central wetland was completed by placing half a meter of clay soil designed to reduce hydraulic conductivity and covered by 0.5–1.0 of salvaged peat obtained from a peatland with both fen and bog site types. The wetland was seeded in winter 2011 with a mix composed largely of *Carex aquatilis* [31]; however, other *Carex* species, including *C. atherodes,* have been growing at the study site (first recorded in 2015). Water from a nearby lake was introduced to the study area in late summer 2012. Currently, the wetland has a nearly complete cover of graminoid vegetation. In 2013, mean Na^+^ concentrations across the entire wetland were at 84.2 ± 7.2 mg L^−1^. Sodium concentrations increased in 2016 to 389 ± 21.7 mg L^−1^, and in 2019 to 494.2 ± 9.7 mg L^−1^. In 2019, sites on the wetland had Na^+^ concentrations as high as 1646.1 mg Na^+^ L^−1^ [36]. In 2017, *Carex aquatilis* dominated large portions of the wetland, with scattered populations of *C. atherodes* [32].

### 4.1. Experimental Design

To determine how *Carex atherodes* responds to increasing levels of sodium, a phytotron experiment was conducted from 24 August 2020 to 11 December 2020 (110 days) using plants grown from seeds collected at Sandhill Wetland (SHW). *Carex atherodes* was exposed to solutions containing one of six sodium concentrations: 60, 789, 1407, 2074, 2363, and 2731 mg Na^+^ L^−1^. We used treatments that contained Na^+^ concentrations naturally occurring in slightly brackish (1), moderately brackish (1), and brackish (4) wetlands (Figure 3). These Na^+^ treatment concentrations are the mean values calculated from the concentrations of Na^+^ utilized in the experiment. The 60 mg Na^+^ L^−1^ treatment was used as a control, representing the absolute highest concentration of Na^+^ expected in natural fens of the region [11].

*Carex atherodes* seeds were collected on 19 September 2019 at SHW. Seeds were wet stratified at 2 °C on moist paper towels, enclosed in plastic bags for 30 days, and germinated on moist peat at 20 °C [38]. Germination occurred four months after stratification. Seedlings (2–4 cm high) were transplanted to pots with a mixture of 1/3 perlite−2/3 vermiculite on 8 July 2020. Each pot was placed in individual polypropylene containers that were filled with a minimum of 400 mL of distilled water. The water contained Jack’s Professional^®^ Water-Soluble Fertilizer (20-3-19 Petunia FeED PlusMg, Allentown, PA; (140.3125 ppm N)) to ensure that the plants had adequate nutrients. Seedlings grew for 47 days after transplant before exposure to sodium treatments, and after excluding the control treatments, subsequently exposed to 1000 mg Na^+^ L^−1^ for one week before treatments.

*Carex aquatilis* seeds were treated in the same manner and grown under treatments of 40, 1035, 1650, 2148, and 2792 mg Na^+^ L^−1^ in order to better define the response between 1056 and 2000 mg Na^+^ L^−1^ [38].

The experiment was performed in the Southern Illinois University Carbondale (37°42’51.9” N 89°13’21.7” W) temperature-controlled phytotron facility with ambient sunlight. Thirty-three *C. atherodes* seedlings were randomly assigned to one of six sodium treatments, each with six replicates (for *C. atherodes,* there were four replicates for the 789 mg Na^+^ L^−1^ treatment, while the 1407 mg Na^+^ L^−1^ treatment had five replicates). In a similar fashion, 30 seedlings of *C. aquatilis* were randomly assigned to one of five sodium treatments, each with six replicates. Individual plants of both species were randomly rearranged and moved to a different location every two weeks. The 18.4 cm round polypropylene containers, each containing one plant, were monitored each week using an electrical conductance (EC) meter to ensure plants were not exposed to higher sodium concentrations. If EC was outside its accepted range (20% higher or lower than its intended sodium concentration in the stock water), the water was emptied and refilled from stock water. Stock water solutions were prepared for each sodium treatment and contained 7 mg L^−1^ magnesium, 4 mg L^−1^ potassium, and 10 mg L^−1^ calcium to mimic natural fen water conditions [38]. Solutions contained 17 L of distilled water, magnesium sulfate (MgSO_4_), potassium bicarbonate (KHCO_3_), calcium oxide (CaO), and sodium sulfate (Na_2_SO_4_) as well as 1/4 teaspoon of the 20-3-19 fertilizer (140.3125 ppm N). Due to calcium oxide’s water-insoluble nature, hydrochloric acid and MES buffer were added to get CaO into the solution in order to bring the stock solution pH to a neutral level (average: 7.38 ± 0.03 S.E.). Sodium was added as a sulfate as it is the dominant anion at Sandhill Wetland [12].

Water samples from the round polypropylene containers were collected each week and concentrations were analyzed for sodium, magnesium, calcium, and potassium on a Varian 220 FS atomic absorption spectrometer; concentrations were also analyzed for EC—using Orion 4 Star EC meter, and for pH—using an Accumet AB15 pH meter. The water in the polypropylene containers was replaced each week with the appropriate stock solutions to maintain treatment water chemistry. To avoid an excessive concentration of sodium in the polypropylene containers, distilled water was used for watering between stock water replacements.

For each *C. atherodes* and *C. aquatilis* plant and ramets produced within each treatment, the following metrics were quantified at the end of the experiment: (1) longest leaf length (sum of longest single leaf from each shoot), (2) number of ramets, (3) chlorophyll content, (4) stomatal conductance, (5) transpiration rate, (6) photosynthesis rate, and (7) dried aboveground and belowground biomass. To estimate the effects of sodium on the amount of chlorophyll present in photosynthetic tissues, a Minolta SPAD 502 chlorophyll meter (Konica Minolta Sensing, Inc., Osaka) was used to measure relative chlorophyll content (no units) at the beginning and end of the experiment after plants reached a sufficient size (3 mm leaf width for *C. atherodes*, 1.5 mm for *C. aquatilis*). A CI-340 handheld photosynthesis system (CID Bio-Science, Inc., Camas, WA) was used to quantify stomatal conductance, transpiration rate, and photosynthesis rate, with three measurements taken for each plant at the end of the experiment (day 100). After the breakdown of the experiment, plants were rinsed with DI water to remove the soil and dried at 60 °C for at least 72 h before measuring aboveground and belowground biomass. Sodium uptake levels in *C. atherodes* roots and shoots were determined using a standard extraction protocol [41].

### 4.2. Statistical Analyses

The responses to sodium concentrations were characterized using an analysis of variance performed in SigmaPlot [42]. For analysis, the six Na^+^ treatment concentrations for *C. atherodes* were 60 (± 1.23) mg L^−1^, 789 (± 71.59) mg L^−1^, 1407 (± 85.66) mg L^−1^, 2074 (± 171.95) mg L^−1^, 2362 (± 97.62) mg L^−1^, and 2731 (± 102.04) mg L^−1^. Similarly, the final Na^+^ treatment concentrations for *C. aquatilis* were 40 mg L^−1^, 1035 mg L^−1^, 1650 mg L^−1^, 2148 mg L^−1^, and 2792 mg L^−1^. The cationic chemistry of the six treatments closely followed the relationship of Na^+^ to EC found at natural brackish and sodic sites (Figure 3), indicating that our treatment chemistry reflected that of natural Na^+^-dominated wetlands.

Physiological responses (stomatal conductance, transpiration rate, photosynthesis rate) of the final month, leaf length (longest leaf length of original plant and longest leaf of any ramets at the end of the experiment), aboveground and belowground biomass, and sodium tissue concentrations were examined for sodium-treatment level differences using a one-way analysis of variance (ANOVA) and Tukey’s pairwise post hoc comparisons when data passed the Shapiro–Wilks test for normality (*p*  <  0.05 failure and visual inspection of residuals). If tests for normality and/or equal variance failed, data were square-root transformed or log transformed. If neither transformation improved normality or equal variance, the untransformed data were analyzed using the Kruskal–Wallis one way analysis of variance on ranks, followed by Dunn’s pairwise post hoc comparison.

## 5. Conclusions

Natural wetland site types with either fresh or brackish porewaters are often habitats for species of *Carex*, including *C. atherodes* and *C. aquatilis*. Both species tolerate porewaters with relatively high concentrations of sodium. Sandhill Wetland currently has many areas with moderately brackish to brackish salinity, including some sites with concentrations up to 1600 mg L^−1^ of sodium. The structural attributes of *C. atherodes* demonstrated reduced performance in phytotron trials above 789 mg Na^+^ L^−1^ and apparent at 1407 mg Na^+^ L^−^^1^, while *C. aquatilis* had a reduced performance above 1650 mg Na^+^ L^−1^ and apparent at 2148 mg Na^+^ L^−1^; these results provide evidence that either of these species tolerate levels of sodium presently recorded at Sandhill Wetland. Although functional attributes for both species become significant at somewhat higher treatments, the large decrease in performance in photosynthesis, transpiration rate, and stomatal conductance in treatments parallel to the structural attributes may suggest responses similar to those in structural attributes. These species, with their high tolerances of brackish concentrations of sodium, may be key ingredients for successful in-pit reclamation designs. The presence of additional high concentrations of divalent cations [43] at reclamation sites may enhance the tolerance of these species to high concentrations of sodium; however, this aspect has not been investigated in wetland species.

## Figures and Tables

**Table 1:** Responses for *Carex aquatilis* to five concentrations of Na^+^.

*Carex aquatilis* Results	40 mg Na^+^ L^−1^	1035 mg Na^+^ L^−1^	1650 mg Na^+^ L^−1^	2148 mg Na^+^ L^−1^	2792 mg Na^+^ L^−1^
Aboveground biomass (g)	2.07 ± 0.18 a	1.49 ± 0.22 ab	1.29 ± 0.13 ab	0.96 ± 0.34 b	0.78 ± 0.17 b
Belowground biomass (g)	2.34 ± 0.23 a	1.60 ± 0.27 ab	1.35 ± 0.31 ab	0.84 ± 0.39 b	0.54 ± 0.09 b
Longest leaf length (cm)	265.88 ± 35.68 a	145.84 ± 15.64 ab	146.38 ± 8.79 ab	91.75 ± 23.84 b	77.83 ± 11.47 b
Ramet count	5.66 ± 1.54 a	3.25 ± 0.55 a	5.67 ± 0.67 a	3.17 ± 0.98 a	3.17 ± 0.83 a
Below:aboveground biomass	1.15 ± 0.08 a	1.04 ± 0.12 a	0.99 ± 0.16 a	0.71 ± 0.18 a	0.75 ± 0.11 a
Na in aboveground biomass (mg g^−1^)	1.57 ± 0.10 a	6.87 ± 0.74 a	9.01 ± 1.04 ab	16.66 ± 2.65 b	15.83 ± 1.95 b
Na in belowground biomass (mg g^−1^)	2.46 ± 0.21 a	8.13 ± 0.49 ab	10.27 ± 1.22 b	13.01 ± 1.57 b	13.50 ± 1.20 b
Na in belowground:aboveground ratio	1.57 ± 0.14 a	1.27 ± 0.10 ab	1.17 ± 0.13 ab	0.91 ± 0.13 b	0.88 ± 0.16 b
Chlorophyll content	34.31 ± 0.60 a	32.64 ± 0.88 a	31.05 ± 1.37 ab	24.66 ± 1.44 c	26.31 ± 1.85 bc
Photosynthetic rate(µmol m^−2^ s^−1^)	7.18 ± 0.82 a	4.52 ± 0.36 ab	3.63 ± 0.55 bc	4.15 ± 0.91 ac	2.35 ± 0.55 c
Transpiration rate(mmol m^−2^ s^−1^)	1.87 ± 0.19 a	0.90 ± 0.05 ab	0.648 ± 0.08 bc	0.46 ± 0.05 c	0.50 ± 0.04 c
Stomatal conductance(mmol m^−2^ s^−1^)	67.85 ± 7.79 a	28.14 ± 1.74 ab	19.96 ± 2.65 bc	15.10 ± 1.81 c	15.85 ± 1.19 c

Values are means ± S.E. Different letters indicate significantly different values between treatments from either Tukey’s pairwise post hoc test at *p* ≤ 0.05 or H = Dunn’s pairwise post hoc comparison at *p* ≤ 0.05). All data from end of experiment.

## Data Availability

Original data are available from the first author upon request and deposited on disc at Southern Illinois University.
